# The intersection of severe mental illness, psychotropic pharmacotherapy, and metabolic disorders

**DOI:** 10.1192/j.eurpsy.2026.10405

**Published:** 2026-07-31

**Authors:** J. Samochowiec, P. Podwalski, B. Dawidowski

**Affiliations:** Psychiatry, Pomeranian Medical University, Szczecin, Poland

## Abstract

**Background:**

Severe mental illness (SMI), including schizophrenia and bipolar disorder, is associated with markedly reduced life expectancy, with premature mortality driven predominantly by preventable somatic comorbidities. Epidemiological data indicate that up to 60% of excess deaths in psychiatric populations are attributable to cardiometabolic disease, including obesity, metabolic syndrome, and type 2 diabetes mellitus (T2DM). Patients with schizophrenia have more than a twofold increased risk of developing T2DM compared with the general population, a vulnerability that emerges even in early, antipsychotic-naïve stages, suggesting shared biological mechanisms beyond lifestyle factors.

**Clinical Problem:**

Psychotropic treatment, particularly second-generation antipsychotics, may further exacerbate metabolic dysregulation through weight gain, insulin resistance, and direct effects on glucose homeostasis. Clozapine and olanzapine are among the most metabolically burdensome agents, whereas partial dopamine agonists such as aripiprazole, brexpiprazole, and cariprazine demonstrate a more favorable metabolic profile. Despite guideline recommendations (APA, NICE, CANMAT), metabolic monitoring and integrated care remain inconsistently implemented.

**Case Presentation:**

We present a clinical case of diabetes decompensation in a patient with SMI receiving long-term antipsychotic therapy, illustrating the complex interplay between psychiatric stability, medication-related metabolic risk, and fragmented somatic care. The case highlights delayed recognition of hyperglycemia, insufficient cardiometabolic screening, and barriers to collaboration between psychiatric and medical services.

**Discussion:**

Emerging evidence supports immunometabolic pathways linking psychiatric disorders with insulin resistance, inflammation (e.g., IL-6), and gut microbiota alterations. Novel interventions, including GLP-1 receptor agonists, show promise in improving glycemic control and weight without worsening psychiatric symptoms.

**Conclusion:**

Metabolic disease in SMI should be considered a core component of psychiatric practice rather than a secondary complication. Integrated dual psychiatric–somatic interventions, systematic screening, and metabolically informed psychopharmacology are essential to reduce preventable morbidity and mortality in this high-risk population.

**Disclosure of Interest:**

None Declared

